# Royal Jelly alleviates sperm toxicity and improves in vitro fertilization outcome in Stanozolol-treated mice

**Published:** 2015-01

**Authors:** Ali Shalizar Jalali, Gholamreza Najafi, Mohammadreza Hosseinchi, Ashkan Sedighnia

**Affiliations:** 1 *Department of Basic Sciences, Faculty of Veterinary Medicine, Urmia University, Urmia, Iran.*; 2 *Department of Basic Sciences, Faculty of Veterinary Medicine, Urmia Branch, Islamic Azad University, Urmia, Iran.*

**Keywords:** *Royal jelly*, *Stanozolol*, *Embryo development*, *Sperm*, *Apoptosis*

## Abstract

**Background:**

Stanozolol (ST) is a synthetic anabolic-androgenic steroid often abused by athletes. An increasing body of evidence points towards the role of ST misuses in the pathogenesis of a wide range of adverse effects including reprotoxicity.

**Objective:**

The aim of this study was to analyze the possible reproprotective effect of royal jelly (RJ) as an efficient antioxidant in ST-treated mice.

**Materials and Methods:**

Adult male mice were divided into four groups (n=5). Two groups of mice received ST (4.6 mg/kg/day) via gavage for 35 days. RJ was given orally to one of these groups at the dose level of 100 mg/kg body weight per day synchronously. Untreated control group and RJ-only treated group were also included. Epididymal sperm characteristics and in vitro fertilizing capacity were evaluated after 35 days.

**Results:**

ST treatment caused a significant (p<0.05) decrease in sperm count and motility and fertilization rate along with poor blastocyst formation and increased sperm DNA damage. Moreover, the incidence of apoptosis and abnormality in spermatozoa was significantly (p<0.05) higher in ST-exposed mice than those of control. The above-mentioned parameters were restored to near normal level by RJ co-administration.

**Conclusion:**

Data from the current study suggest that RJ has a potential repro-protective action against ST-induced reproductive toxicity in mice. However, clinical studies are warranted to investigate such an effect in human subjects.

## Introduction

Widespread abuse of anabolic-androgenic steroids (AASs) has caused great concern because of the large adverse systemic and psychological effects of these compounds (1). There is evidence that 3-12% and 1-2% of adolescent males and females living in the Western world admit to the former or present non-medical use of AASs, respectively (2). Despite the use of AASs being prohibited in the organized sports, their abuse is also found in over 50% of positive doping cases (3). 

Although, AASs have established their efficacy in treating anemias, osteoporosis, muscle-wasting conditions and AIDS-related cachexia, a growing body of evidence indicates that supraphysiological doses of AASs are associated with psychiatric, cardiovascular, hepatic, endocrine and reproductive disorders (4-11). Consistent with these reports, it has been proven that stanozolol (ST) as an active nutritional 17α-alkylated AAS causes endocrine system dysfunction and reproductive toxicities in humans and experimental animals (12-14).

On the other hand, accumulating evidence demonstrates the effectiveness of natural remedies with antioxidant and anti-inflammatory activities against drug-induced reproductive toxicities (15-17). Royal jelly (RJ) a secretion product of the cephalic glands of worker honeybees, is a highly efficient antioxidant and possesses prominent free radical scavenging property (18, 19). Moreover, multiple independent studies have emphasized that in addition to the antioxidant action, RJ has pleiotropic effects, including its beneficial antitumor, antibiotic, immunomodulatory and anti-inflammatory activities (20-23).

Although previous reports have indicated that RJ has significant positive effects on the reproductive system and fertility, less attention has been directed towards disclosing its potential in protection against drug-induced sperm apoptosis and embryo toxicity (24, 25). In line with that, the objective of this study was to elucidate the possible repro-protective effect of RJ against reproductive toxicity caused by ST exposure in mice.

## Materials and methods

**Animals**

In this experimental study, 20 healthy adult male Naval Medical Research Institute (NMRI) mice, weighing 23±1.1 gr, were obtained from the Animal House, Faculty of Veterinary Medicine, Urmia University, Urmia, Iran. The animals were placed in clean polycarbonate cages in a specific pathogen-free and well-ventilated room. Animal room temperature and relative humidity controls were set at 22±2^o^C and 50±10%, respectively. Lighting was controlled to give 12 hr light and dark cycles. The animals were supplied with standard laboratory chow and tap water ad libitum throughout the experiment. The experimental procedures were carried out in accordance with international guidelines for care and use of laboratory animals and approved by the Ethical Committee of Urmia University.

**Treatment**

After 1-week acclimatization, the mice were randomly assigned to four equal groups each comprising five animals and treated orally for 35 days as follows: 1) untreated control, 2) treated with ST (4.6 mg/kg/day), 3) treated with RJ alone (100 mg/kg/day) and 4) treated with RJ (100 mg/kg/day)+ ST (4.6 mg/kg/day). The protocol for this study, including doses and duration of treatment for ST and RJ, were all designed according to previous studies (26, 27).

**Sampling**

Animals were euthanized following anesthesia with a mixture of ketamine (45 mg/kg; IP) and xylazine (35 mg/kg; IP) 24 hr after the final treatment. A vertical midline lower abdominal incision was made and epididymides were carefully dissected out and cleaned of adhering connective tissue under a 20-time magnification provided by a stereo zoom microscope (Model TL2, Olympus Co., Tokyo, Japan).

**Sperm characteristics**

Epididymal sperms were collected by chopping one caudal epididymis in 1 ml of human tubular fluid (HTF) solution and incubated for 10 minutes at 37^o^C in an atmosphere of 5% CO_2_ incubator to allow sperm to swim out of the epididymal tubules. In order to assess the sperm motility, one drop of sperm suspension was placed on a microscope slide, and a cover slip was placed over the droplet. At least 10 microscopic fields were observed at 400x magnification using a phase contrast microscope, and immotile sperms percentages were calculated (28).

The epididymal sperm counts were obtained by the standard hemocytometric method as described previously (29). Briefly, after dilution of epididymal sperm to 1:20 in HTF medium, approximately 10 μl of this diluted specimen was transferred to each of the counting chambers of the hemocytometer, which was allowed to stand for 5 minutes in a humid chamber to prevent drying. The cells sedimented during this time and were counted with a light microscope at 400×. The sperm count was expressed as number of sperm per milliliter. 

Acridine orange (AO) staining was used to assess the cauda epididymal sperm DNA denaturation. For the analysis of sperm DNA integrity with fluorescence microscopy, thick smears were fixed in Carnoy’s fixative (methanol: acetic acid 1: 3) for at least 2 hours. The slides were stained for 5 minutes and gently rinsed with deionized water. 200 sperms were evaluated and sperm heads with denatured chromatin displayed an orange-red fluorescence when compared to those with intact chromatin which emitted green fluorescence (30). 

Evaluation of sperm abnormalities was performed as described previously (31). Sperm smears were prepared on clean and grease free slides, allowed to air-dry overnight, stained with 1% eosin-Y/5% nigrosin and examined at 400x magnification for morphological abnormalities. Teratozoospermia index (TZI) is defined as the number of abnormalities present per abnormal spermatozoon. Each abnormal spermatozoon can have one to four abnormalities, including head, neck/midpiece and tail defects or presence of cytoplasmic residues. The spermatozoa are recorded as normal or abnormal and distributed into specific groups (head, neck/midpiece and tail defects or cytoplasmic residues groups). The total number of abnormalities is then added together and divided by the number of abnormal spermatozoa (32).

**Quantification of sperm apoptosis**

Phosphatidylserine (PS) externalization following lipid remodeling of the sperm plasma membrane is a primary feature of apoptosis (33). To detect PS translocation, the Annexin-V FITC Apoptosis Detection Kit was used (BD Biosciences, Pharmingen, San Diego, USA). Annexin V, an anticoagulant protein, binds outwardly exposed PS with high affinity in a calcium-dependent manner (34). To perform this assay semen samples containing 1×10^6^ spermatozoa were centrifuged at 5000 rpm for 6 min and resuspended in equal volume of HEPES-buffered saline. Semen suspension was mixed with 100 μl annexin-V/fluorescein isothiocyanate solution and incubated for 15 min at room temperature. Staining with annexin V was checked under fluorescent microscope using 488 nm wave-length filter. Sperm with disordered membrane exhibited green fluorescence, whilst live sperm was unstained. Apoptotic index (AI) was defined as the number of apoptotic annexin V-positive sperm cells per 100 spermatozoa (35)

**Ovulation induction and oocyte collection**

To induce superovulation, female mice were injected intraperitoneally with 7.5 IU of pregnant mare’s serum gonadotropin (PMSG, Folligon, the Netherlands) and 7.5 IU of human chorionic gonadotropin (hCG, Folligon, the Netherlands) 48 hr later (36). 14 hr after hCG administration, females were sacrificed by cervical dislocation following anesthesia with ketamine (75 mg/kg; IP). Their oviducts immediately excised and placed in Petri dishes containing HTF medium. Using a stereo zoom microscope (Model TL2, Olympus Co., Tokyo, Japan), the ampullary portion was found and oocytes were removed.

**In vitro fertilization**

Spermatozoa were derived from cauda epididymis as described earlier and preincubated for 1 hour in HTF medium at 37^o^C in an atmosphere of 5% CO_2_ to ensure capacitation. A volume of 0.1 mL of sperm suspension was introduced into 0.9 mL fertilization drop of oocytes-containing HTF medium. For each animal, a total of 20 oocytes were divided into 10 drops. After six hours of incubation at 37^o^C under 5% CO_2_, the cumulus cell free fertilized oocytes were transferred to fresh drops of HTF medium for culture of embryos. All fertilization steps and embryo culture were carried out under detoxified mineral oil (37).

**Microscopic evaluations**

24 hr after insemination, oocytes were evaluated via an inverted microscope under 200x magnifications and the percentage of oocytes with 2 pronuclei was recorded to determine in vitro fertilization success rate (ISR) (38). Blastulation rate (BD) and hatching rate (HR) were calculated by determining the number of embryos that had reached the blastocyst development stage and the number of fully hatched blastocysts after 72 hr incubation, respectively (39, 40).

**Statistical analysis**

The data were expressed as mean±SE. The variables were analyzed by one-way analysis of variance followed by Tukey test for post hoc comparisons using Statistical Package for the Social Sciences, version 18.0, SPSS Inc., Chicago, Illinois, USA. The statistical significance level was set at p<0.05.

## Results

**Epididymal sperm parameters**

Table I shows the effect of ST and RJ treatment on epididymal sperm characteristics. As it is clear from the table, epididymal sperm concentration and motility were significantly lower in mice treated with ST in comparison to control and RJ alone animals. Fascinatingly, administration of RJ to ST-treated mice resulted in a nearly complete reversal of the ST-induced decreases in epididymal sperm count and motility to the normal values. The results of AO staining showed that the mean percentage of spermatozoa with DNA damage increased significantly in ST-treated mice when compared to control mice. Interestingly, administration of RJ in combination with ST markedly impeded the ST-induced increase in the epididymal sperm DNA damage (Table I). In the mice subjected to ST treatment, a statistically significant elevation of TZI value was observed as compared to control mice, whereas RJ treatment along with ST provided marked normalization in the TZI value when compared to the ST-only treated group (Table I). 

**Sperm apoptosis**

**Figure 1:**
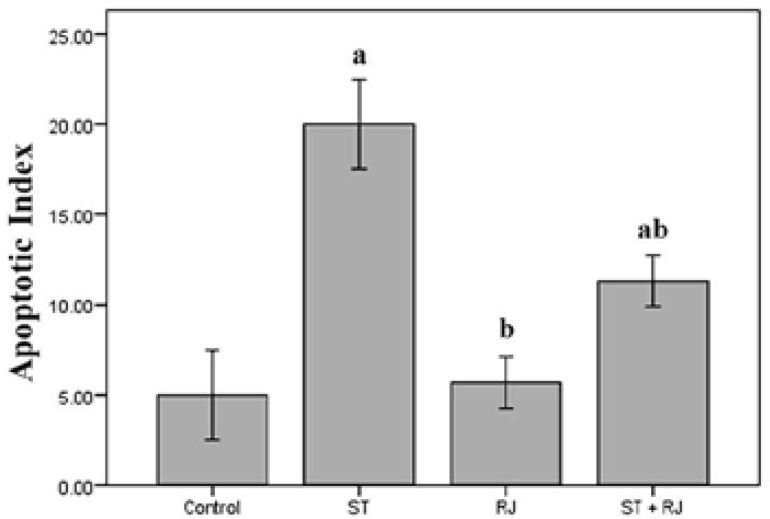
Effect of stanozolol and royal jelly treatment on sperm apoptosis in mature male mice. Histogram represents apoptotic index expressed as apoptotic sperm cells per 100 spermatozoa.

ST, Stanozolol; RJ, Royal jelly. The values are expressed as mean±SEM (n= 5).

^a ^p<0.05 versus control group.

^b ^p<0.05 versus stanozolol group.

Figure 1 represents data concerning sperm cell apoptosis in all experimental groups. As expected, AI was significantly higher in the ST group versus the control and RJ alone groups, whilst RJ co-administration substantially reduced AI as compared to the ST group non-treated with RJ.

**In vitro fertilization outcome**

**Figure 2:**
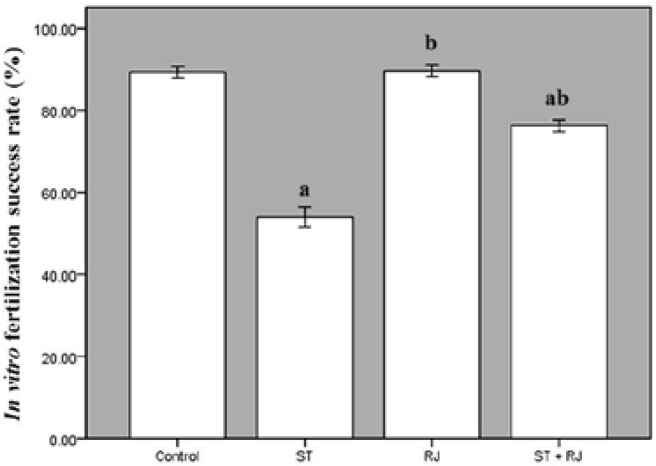
Effect of stanozolol and royal jelly treatment on in vitro fertilization success rate.

ST, Stanozolol; RJ, Royal jelly. The values are expressed as mean±SEM (n= 5).

^a ^p<0.05 versus control group.

^b ^p<0.05 versus stanozolol group.

**Figure 3:**
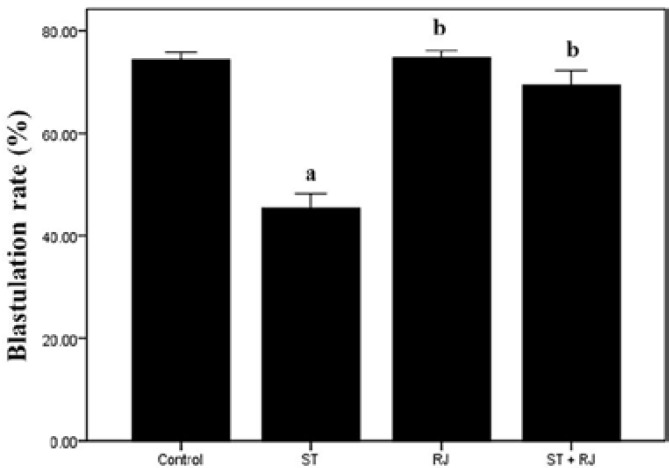
Effect of stanozolol and royal jelly treatment on the rate of blastulation.

ST, Stanozolol; RJ, Royal jelly. The values are expressed as mean±SEM (n= 5).

^a ^p<0.05 versus control group .

^b ^p<0.05 versus stanozolol group.

**Figure 4:**
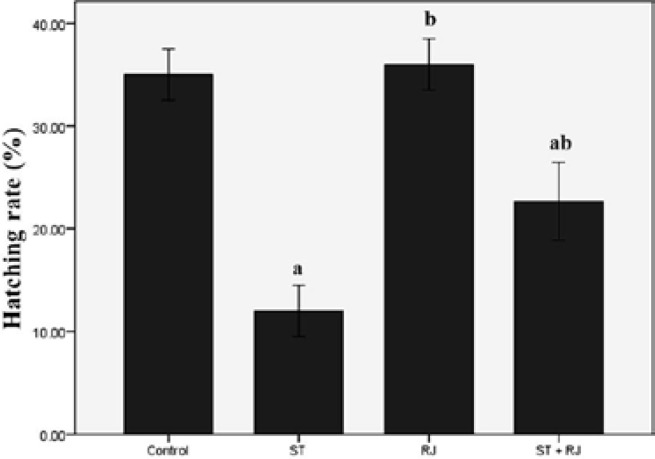
Effect of stanozolol and royal jelly treatment on the rate of blastocyst hatching.

ST, Stanozolol; RJ, Royal jelly. The values are expressed as mean±SEM (n= 5).

^a ^p<0.05 versus control group .

^b^p<0.05 versus stanozolol group.

Figure 2 compares ISR among the four experimental groups. The proportion of fertilized oocytes to inseminated oocytes was significantly lower in ST-only treated mice than in the control group. Co-treatment with RJ resulted in a significant improvement in this parameter compared to the ST alone group. The effects of different treatments on BD are depicted in Figure 3. The proportion of embryos that became blastocysts in ST alone group was significantly lower than that in the control group. Mice treated with RJ simultaneously exhibited amelioration of ST-induced embryo toxicity. Figure 4 summarizes the effects of ST and RJ on HR. The rate of completely hatched blastocysts was significantly smaller in ST-received mice compared to that in the controls, while this ratio was nearly restored to control levels by RJ co-administration.

**Table I:** Effect of stanozolol and royal jelly treatment on epididymal sperm characteristics

	**Control**	**ST**	**RJ**	**ST + RJ**
Sperm count (10^6^/ml)	38.06 ± 1.01	23.66 ± 1.45^a^	42.00 ± 2.30^b^	34.99 ± 2.15^b^
Immotile sperms (%)	8.66 ± 0.88	18.66 ± 0.88^a^	9.00 ± 0.57^b^	13.00 ± 1.52^b^
Positive AO staining (%)	16.33 ± 0.88	24.33 ± 0.88^a^	15.66 ± 0.88^b^	18.66 ± 0.88^b^
TZI	1.16 ± 0.07	1.90 ± 0.05^a^	1.20 ± 0.07^b^	1.41 ± 0.02^a^^b^

ST: Stanozolol

RJ: Royal jelly

AO: Acridine orange

TZI: Teratozoospermia index.

The values are expressed as mean ± SEM (n= 5).

a Significant differences as compared with the control group at p<0.05.

b Significant differences as compared with the stanozolol group at p<0.05.

## Discussion

The abuse of AASs has been the focus of researches worldwide, since these compounds administration is often associated with various dose- and time-dependent side effects including male reproductive health deterioration (41). Although the precise pathological mechanism underlying AASs-induced male reproductive toxicities have not yet been completely elucidated, AASs have been found to induce hypogonadotrophic hypogonadism coupled with decreased total and free testosterone circulating levels via alterations in the hypothalamic-pituitary-gonadal axis (42, 43). In the present study, male mice exposed to ST showed impaired sperm parameters. These results confirm and extend previous data which have demonstrated that AASs induce severe oligospermia and azoospermia and are spermatotoxic (44, 45).

It is well known that spermatogenesis is a highly complicated process in which immature male germ cells (MGCs) differentiate into mature spermatozoa (46). Sertoli cell as a specialized somatic cell play a pivotal role in survival, proliferation and maturation of MGCs (47). Moreover, AASs have been reported to induce marked depressions of serum testosterone and sex hormone-binding globulin through androgenic receptors occupation (48). Therefore, spermiogenesis failure in the ST-treated mice may be explained by the disruption of testosterone-dependent attachment of MGCs to Sertoli cells.

On the other hand, it has been clearly described that ST administration provokes mitochondriopathy, resulting in an enhanced generation of reactive oxygen species (ROS) and a state of oxidative stress (OS) (49). There is overwhelming evidence that OS can cause sperm dysfunction through different mechanisms which include lipid peroxidation of the sperm plasma membrane, impairment of sperm motility and morphology and induction of high frequencies of single and double DNA strand breaks (50, 51). Further, the existing literature indicates that OS-induced sperm DNA damage accelerates the process of germ cell apoptosis leading to the decline in sperm concentrations (52). Supporting these facts, our findings suggest that ST treatment causes high levels of DNA damage and apoptosis in spermatozoa via mechanisms that may be mediated by OS. Previous reports point to the possible relationship between seminal oxidative stress and defective embryo development and retardation of embryo growth (53, 54). 

In addition, it has been demonstrated that sperm DNA damage leads to decreased fertilization rates and/or embryo cleavage (55). In light of the fact that structurally abnormal spermatozoa are the major source of ROS production in semen, ISR, BD and HR reduction in ST-administered mice may be attributed to the injurious effects of ROS-producing damaged spermatozoa during in vitro insemination of oocytes (56). Recently, there have been increasing interests in the search of potential compounds that are capable of minimizing the chemical-induced reproductive toxicities. Several reports have revealed that RJ possesses a variety of biological activities, including anti-oxidative, anti-inflammatory and repro-protective effects (22, 57, 58). 

In the current study, concomitant administration of RJ to ST receiving mice noticeably improved the ST-induced all the negative changes observed in the sperm parameters and embryo development. The protection offered by RJ against ST reprotoxicity is likely thanks to its ability to inhibit OS by neutralizing ROS as well as its interaction with androgen receptors that are occupied by ST. Similar to our observations, it has been shown that RJ has high anti-oxidative activity and scavenging ability against free radicals and can help to prevent cisplatin-induced spermiotoxicity in adult male rats (58). In the same concern, it was also found that RJ counteracts “summer infertility” in male rabbits (59). 

Our recent study has also revealed that RJ can effectively attenuate AASs-induced oxidative injury in mouse testis through restoration of antioxidant defense system (60). Collectively, in investigating the potential protective effects of RJ in OS- mediated reprotoxicity arising as a result of ST administration in mice, RJ through its antioxidant activity provides a significant protective effect against sperm impairment and embryo toxicity. Further studies will be required to assess the applicability of RJ in clinical trials. 

## Conflict of interest

The authors declare no potential conflicts of interest with respect to the authorship and publication of this article.
